# Examining the Nutrition of Cardiological Patients in Hospitals: Evaluating the Discrepancy between Received Diets and Reference Diet Based on ESC 2021 Guidelines—Hospital Diet Medical Investigation) (HDMI) Study

**DOI:** 10.3390/nu15214606

**Published:** 2023-10-30

**Authors:** Daniel Śliż, Alicja Monika Jodczyk, Klaudia Łakoma, Alicja Kucharska, Mariusz Panczyk, Olga Maria Rostkowska, Karolina Turlej, Agnieszka Młynarska, Jarosław Drożdż, Milena Jarzębska-Wódka, Piotr Wierzbiński, Marcin Grabowski, Anna Ukleja, Natalia Adamczyk, Alicja Baska, Szczepan Wiecha, Marcin Barylski, Adam Rafał Poliwczak, Artur Mamcarz

**Affiliations:** 1 3rd Department of Internal Medicine and Cardiology, Medical University of Warsaw, 04-749 Warsaw, Poland; 2 Polish Society of Lifestyle Medicine, 00-388 Warsaw, Poland; 3 Department of Human Nutrition, Medical University of Warsaw, 01-445 Warsaw, Poland; 4 Department of Education and Research in Health Sciences, Medical University of Warsaw, 00-518 Warsaw, Poland; 5 Department of Transplantation Medicine, Nephrology and Internal Medicine, Medical University of Warsaw, 02-006 Warsaw, Poland; 6 Department of Gerontology and Geriatric Nursing, Medical University of Silesia, 40-635 Katowice, Poland; 7 Department of Cardiology, Medical University of Lodz, 92-213 Lodz, Poland; 8 1st Chair and Department and of Cardiology Medical University of Warsaw, 02-097 Warsaw, Poland; 9 School of Public Health, Centre of Postgraduate Medical Education, 01-813 Warsaw, Poland; 10 Department of Physical Education and Health in Biala Podlaska, Faculty in Biala Podlaska, Jozef Pilusdski University of Physical Education, 00-968 Warsaw, Poland; 11 Department of Internal Medicine and Cardiac Rehabilitation, Medical University of Lodz, 90-647 Lodz, Poland

**Keywords:** cardiology, nutrition, hospital, diet

## Abstract

Cardiovascular diseases (CVDs) are the leading causes of death worldwide. CVDs have become the dominant cause of death and have been a significant health challenge since the second half of the 20th century in the Polish population. The aim of our HDMI (hospital diet medical investigation) study was to examine the quality of the hospital diets given to cardiac patients and assess how much they adhere to the European Society of Cardiology (ESC) 2021 guidelines. By comparing the diets received by patients with the recommended dietary patterns outlined in the ESC 2021 guidelines, we sought to identify discrepancies. The study was conducted in two steps: creating a 7-day model menu and comparing it with the received diets and then making comparisons with ESC 2021 guidelines. Additionally, we designed a survey to obtain the characteristics of the hospitals. The results show that the nutrition in hospitals remains substandard. None of the diets had an appropriate salt supply or predominance of plant-based food patterns. Only 1/7 diets avoided sweetened beverages, and 2/7 diets had an appropriate amount of fiber. This underscores a gap in the healthcare system to improve patients’ health by implementing dietary interventions that foster the development of healthy eating habits.

## 1. Introduction

Cardiovascular diseases (CVDs) are a leading cause of mortality worldwide, requiring specialized care for cardiological patients, leading significantly to excessive costs for the healthcare system [1,2,3]. In Poland, CVDs have been a major health problem since the second half of the 20th century, and they remain the main cause of death among Polish residents [4,5]. In 2019, CVDs accounted for 39.4% of deaths, but due to the COVID-19 pandemic in 2021, the data show that they decreased to 34.8% [4].

Proper nutrition plays a pivotal role in managing and treating these patients, influencing their recovery and long-term outcomes [1]. Hospitalized cardiac patients often rely on dietary interventions provided by healthcare facilities to aid their recovery and overall cardiovascular health. It should be pointed out that the patient’s poor nutritional status in hospitals reduces the effectiveness of their treatment and increases the risk of complications [6]. It has already been proven that targeted nutritional intervention can prevent unplanned readmissions [7]. Moreover, it has already been shown that the patient’s nutritional education starts in the hospital and is crucial in improving lifestyle and well-being [8]. For this reason, the importance of evidence-based healthcare including nutrition care in a hospital setting is emphasized [1]. Promoting healthy dietary habits is a crucial aspect of patient care that extends beyond the hospital. It is essential to underscore the pivotal role that a patient’s daily dietary choices and lifestyle play in their overall well-being. Notably, the prevailing diet among the typical Polish population leans more toward a Western dietary pattern than the healthier Mediterranean diet.

It is noteworthy that a mere 24% of the Polish population can be classified as adhering to a wholesome diet, while the majority of individuals encounter various hurdles. Alarmingly, 29.1% openly express their reluctance to embrace healthier eating habits. These statistics strongly underscore the imperative need for healthcare professionals to step in and provide guidance and intervention [4]. Regrettably, nutritional and dietary interventions still tend to be undervalued in the routine clinical management of chronic diseases [9].

In the case of cardiac diseases, proper nutrition constitutes not only an element of prevention but also an essential part of treatment, as confirmed by the latest 2021 European Society of Cardiology (ESC 2021) recommendations [10]. However, the extent to which these hospital diets align with the latest guidelines remains unclear. In addition, in 2018, the Supreme Audit Office alerted that diets in Polish hospitals were poorly balanced [11]. Only 17% of hospitals examined in this report had an appropriate caloric value. In addition, the diets lacked important nutrients such as calcium, iron, magnesium and potassium. Deficiencies of the above ingredients were accompanied by an increased supply of salt (142–374% of the norm) and vitamin A. The meals provided in the hospitals did not fulfill their essential function—supporting the treatment and recovery process—and sometimes could constitute a harmful factor. It was highlighted that not only a well-planned diet but also the support of a dietician was indicated as the key factor determining the improvement in the patient’s health. The existing gap within the healthcare system was identified in the form of inadequate hospital nutrition that failed to meet the patient’s specific needs. It was also emphasized that, ultimately, hospital nutrition frequently fell short in contributing to the improvement in the patient’s health [11]. Given the lapse of five years since the issuance of the 2018 report, our pilot study tries to meticulously assess the quality of hospital diets, thus making a comprehensive comparison with the current ESC guidelines in the context of a cardiac patient.

This publication is part of an HDMI (hospital diet medical investigation) study and aims to investigate the nutrition provided to cardiological patients in hospitals and assess how much it adheres to the ESC 2021 guidelines. By comparing the actual diets received by patients with the recommended dietary patterns outlined in the ESC 2021 guidelines, we seek to identify any gaps and discrepancies. Understanding the existing disparities between received diets and evidence-based recommendations is crucial for optimizing the care and outcomes of cardiological patients.

A healthy diet is recognized as a fundamental pillar of CVD prevention, with an emphasis on several key principles that include a focus on plant-based foods, the limitation of saturated fatty acids to less than 10% of energy intake, aiming for a daily fiber intake of 30–45 g, incorporating fish into the diet 1–2 times per week, restricting salt intake to less than 5 g per day and limiting the consumption of alcohol, red meat and sugary beverages [10]. A randomized, single-blind secondary prevention trial, the Lyon Diet Heart Study, showed that the Mediterranean diet reduced the recurrence rates after the first myocardial infarction compared to a prudent Western-type diet. Patients randomized to the Mediterranean diet experienced a 72% reduction in recurrent non-fatal myocardial infarction and a 56% decline in mortality compared to the control diet [12]. One of the biggest challenges in the dietary prevention of CVDs lies in devising more effective strategies to motivate individuals to initiate dietary changes and, equally importantly, sustain them over time [13].

We hypothesized that the diet served to cardiological patients in Polish hospitals did not fulfill the ESC 2021 recommendations and was not properly balanced.

## 2. Materials and Methods

### 2.1. Design and Setting

Our comparative cross-sectional study was conducted between January and August 2022. The study was carried out in four stages: creating a survey, preparing the reference diet, evaluating diets in “Dieta 6” computer program and comparing the results with ESC recommendations.

### 2.2. Sample

Hospital workers (heads of the departments, specialists and resident doctors) from various districts in Poland were invited to participate in a study through email, which contained information about the purpose of the study, terms of the participation and required information. To qualify for participation, hospitals had to be public, have a general medicine ward and complete the questionnaire. Private hospitals or hospitals specializing in certain illnesses or groups of patients, such as military personnel, were excluded. Filling in the questionnaire and clicking the “send” button were tantamount to informed consent to participate in the study (proper information was mentioned in the background of the questionnaire).

### 2.3. Bioethical Consideration

The study adheres to the regulations set by the Institutional Ethics Committee of the Medical University of Warsaw and aligns with the principles outlined in the Helsinki Declaration (1964). It was not necessary to acquire approval from the Institutional Review Board, but a thorough assessment was obtained. Respondents did not receive any monetary or material rewards for their participation. Anonymity was emphasized at the beginning of the questionnaire, along with a comprehensive explanation of the study’s objectives.

### 2.4. Instrument and Data Collection

The study involved (1) a survey with questions about general information and the organization of hospital food service within the hospital, as well as (2) submitting menus of patient meals for the following 10 days. The self-made questionnaire, created using Google Forms, consisted of 12 questions. It included information about the hospital’s degree of reference (1st/2nd/3rd degree of reference—the division is based on the specific regulations in Poland and classifies hospitals by the number of beds, the competence of medical personnel, access to equipment, diagnostic and therapeutic procedures; 1st is the lowest degree with limited and basic medical procedures, and 3rd is the highest with the most extended and specialized procedures), number of beds in total, internal and cardiology unit, presence of a person providing nutritional education (nurse/physician/dietician), location of meal preparation (hospital kitchen/catering) and availability of special diet dedicated for patients with cardiological problems. The data collection period was from 26th January 2022 to 3rd August 2022. Data sent via Google Forms and email were exported as an Excel file for further analysis. The questionnaire is attached in the Supplementary S1.

The received menus were evaluated using “Dieta 6”, which is a unique computer program standardized for the Polish population. It was designed by the National Institute of Public Health—National Institute of Hygiene in Warsaw. It was based on national composition tables and the nutritional value of food products and meals. It enables researchers to calculate the average nutritional value and composition of the consumed diet.

We created a model seven-day menu (standardized menu) to compare different hospital menus with the European Society of Cardiology (ESC) recommendations [10]. We aimed to create a genuine and achievable diet with the inclusion of universal, widely available and relatively low-cost ingredients. This menu is attached in the Supplementary S2. The guidelines were published in 2021 [10] to assist healthcare professionals in reducing the burden of CVD on both individual patients and the population. They emphasize the importance of educating patients (class IIa according to ESC 2021 recommendations), intervening in CVD risk factors and promoting a healthy diet. Table 1, based on the guidelines, outlines the main features of a healthy diet, which is recommended for patients with cardiological problems.

### 2.5. Statistical Analysis

Statistical analyses were conducted utilizing STATISTICA™ 13.3 software (TIBCO Software, Palo Alto, CA, USA). Nutritional values were computed from menu data using DIETA Software version 6.0 (National Institute of Public Health—National Institute of Hygiene in Warsaw). Descriptive statistics, including mean (M), standard deviation (SD) and range (Min–Max), were calculated for each nutritional value. One-way ANOVA with post hoc Dunnett’s two-tailed test was employed to assess the differences in means for nutrients and products between hospitals and the standardized menu. Dunnett’s test is a multiple comparison procedure that compares each of several means with a single control in many-to-one comparisons. The null hypothesis was rejected for *p*-values less than 0.05, indicating that the tested difference in means was statistically significant.

## 3. Results

### 3.1. The Outcomes from the Survey

The study involved seven hospitals, out of which five were classified as third degree of reference, one as second degree and one as first degree. While six hospitals had a separate cardiology ward, only Hospital 5 provided a specific diet for cardiological patients. Nutrition education was provided in six hospitals, with only four having a dietician present. In terms of meal preparation, four hospitals prepared their own meals, while three ordered catering services. Table 2 displays the general characteristics of the hospitals, such as hospital type, bed capacity (total, internal medicine and cardiology unit), availability of nutrition education and meal preparation location. For further analysis, due to the lack of a specific cardiological diet, the general diet from Hospitals 1, 2, 3, 4, 6 and 7 was taken into consideration. From Hospital 5, a cardiological diet was examined.

### 3.2. Differences in the Content of Nutrients and product Groups

Table 3 demonstrates energy and carbohydrate content. A one-way ANOVA revealed that there was a statistically significant difference in the mean of energy (kcal) (F = 12307.61; *p* < 0.001), the mean of total carbohydrates (F = 14789.56; *p* < 0.001), the mean percentage of energy from carbohydrates (F = 16905.28; *p* < 0.001), the mean percentage of digestible carbohydrates (F = 13424.08; *p* < 0.001) and the mean percentage of fiber (F = 5870.27; *p* < 0.001) between hospitals. Compared to the standardized menu, there was a statistically greater mean of energy in Hospital 2 (*p* = 0.001), a greater mean of total carbohydrates in Hospitals 2 (*p* = 0.041), 6 (*p* < 0.001) and 7 (*p* = 0.001), a greater mean percentage of energy from carbohydrates in Hospitals 1 (*p* = 0.012), 3 (*p* = 0.005), 5 (*p* = 0.001), 6 (*p* < 0.001) and 7 (*p* < 0.001), a greater mean of digestive carbohydrates in Hospitals 1 (*p* = 0.001), 2 (*p* = 0.001), 6 (*p* < 0.001) and 7 (*p* < 0.001) and a lower mean of fiber in all hospitals (*p* < 0.001).

Table 4 illustrates the protein content of the hospitals. A statistically significant difference was observed between hospitals in the mean of total protein (F = 7647.82; *p* < 0.001), the mean percentage of energy from protein (F = 6927.64; *p* < 0.001), the mean of plant-based protein (F = 5740.80; *p* < 0.001) and the mean of animal-based protein (F = 2150.84; *p* < 0.001). Compared to the standardized menu, Hospitals 1, 3, 4, 5, 6 and 7 had a lower mean of total protein (*p* < 0.001), a lower mean percentage of energy from protein (*p* < 0.001 for Hospitals 1, 2, 3, 5, 6 and 7, and *p* = 0.003 for Hospital 4) and a lower mean of plant-based protein (*p* < 0.001). Hospital 2 had a significantly higher mean of animal-based protein (*p* = 0.007).

Table 5 displays the fatty acid content, and there were significant variations among hospitals in the mean of total fat (F = 1697.67; *p* < 0.001 *), the mean percentage of energy from fat (F = 3635.99; *p* < 0.001 *), the mean percentage of saturated fatty acids (F = 2027.01; *p* < 0.001 *), the mean percentage of polyunsaturated fatty acids (F = 1000.07; *p* < 0.001 *), the mean percentage of *n*-3 acids (F = 328.40; *p* < 0.001 *) and the mean percentage of *n*-6 acids (F = 1251.11; *p* < 0.001 *). Compared to the standardized menu, Hospital 2 had a significantly greater mean of total fat content (*p* = 0.011). The variation in the mean percentage of energy from fat was not statistically significant (*p* > 0.05). All hospitals except Hospital 4 had a greater mean of saturated fatty acids (*p* < 0.001), and each hospital had a lower mean of polyunsaturated fatty acids (*p* < 0.001) and *n*-6 acids (*p* < 0.001). Hospital 5 had a lower mean of *n*-3 acids (*p* = 0.022).

The mean of sodium and salt intake varied among the hospitals (F = 3411.58, *p* < 0.001, and F = 3416.12, *p* < 0.001), but the difference was not statistically significant (*p* > 0.05). Precise values are presented in Table 6.

The hospitals showed significant differences in the mean mass of fruits (F = 723.54; *p* < 0.001 *), the mean mass of vegetables (F = 976.51; *p* < 0.001 *), the mean mass of whole-grain products (F = 65.67; *p* < 0.001 *), the mean mass of processed meat (F = 717.13; *p* < 0.001 *) and the mean mass of sugar (F = 1327.33; *p* < 0.001 *). A lower mean mass of fruits was provided in Hospital 1 (*p* = 0.022) and Hospitals 3, 4, 5, 6 and 7 (*p* < 0.001). A lower mean mass of vegetables was provided in Hospitals 1, 3, 4, 5, 6 and 7 (*p* < 0.001). A lower mean mass of whole-grain products was provided in Hospitals 1 (*p* = 0.007), 3 (*p* = 0.012), 6 (*p* = 0.002) and 7 (*p* = 0.002). Due to shortages in particular menus, an analysis of legumes, nuts and beans, fish and nuts was not conducted. Every hospital provided red meat in their diet and the reference diet did not. Fish was provided only in one meal once a week in most of the hospitals. Nuts and beans were provided only in Hospitals 1, 3 and 4. Precise values are presented in Table 7.

### 3.3. Fulfilling the ESC Guidelines

The hospitals were evaluated based on the ESC recommendations [10] mentioned earlier, and the findings are reported in Table 8. All hospitals successfully met four of the criteria, which included intake of vegetables and fish, reduced consumption of red meat and avoidance of alcohol. However, none of the hospitals provided a diet that predominantly consisted of plant-based food and limited consumption of animal-based food, ensured intake of unsalted nuts or limited salt intake to less than 5 g. Hospital 4 met six out of eleven criteria, while Hospitals 2, 3 and 5 met five out of eleven criteria. Hospitals 1, 6 and 7 could only fulfill four out of eleven criteria.

### 3.4. Estimated Number of Patients

Each hospital has a specific number of beds, taking into account the province in which it is located, and there is an annual occupancy rate. Based on these factors, it is possible to estimate the total number of patient beds and person days of hospitalization. For example, Hospital 1 has 1035 beds, Hospital 2 has 452 beds, Hospital 3 has 683 beds, Hospital 4 has 677 beds, Hospital 5 has 363 beds, Hospital 6 has 176 beds, and Hospital 7 has 321 beds. By summing up these data, we can determine the number of cardiological patients who represent a missed opportunity for nutrition education, ultimately having a negative impact on the healthcare system. Hospitals 1, 2 and 3 are in the Mazowieckie Voivodeship, where there is an average of 40.8 beds per year, which gives us (2170 × 40.8) 88536patients. Hospitals 4, 6 and 7 are in the Śląskie Voivodeship, where there is an average of 36.2 beds per year, i.e., (1174 × 36.2) 42498,8patients, and Hospital 5 is in the Łódzkie Voivodeship, where the index is 42.4, which means approx. 15391 patients per year [14]. Adding up the number of patients, we get an estimated result: 146425,8patients per year.

## 4. Discussion

### 4.1. A Summary of Main Results

Based on the data mentioned above, it is crucial to acknowledge that despite the ESC recommendations with the latest introduced in 2021 [10], there remains a significant oversight in incorporating them into dietary planning in hospitals, consequently having an adverse impact on the health of cardiac patients. The collected data highlight a substantial disparity within the Polish healthcare system. Firstly, certain hospitals do not provide specialized cardiological diets, instead offering general diets that fail to meet the specific nutritional requirements and deviate significantly from the proposed standards. Secondly, hospitals should serve as an environment for initiating nutrition education, exposing patients to well-balanced diets that acquaint them with heart-healthy eating habits. However, the absence of a standardized dietary model poses a risk of patients making nutritional errors that may have a detrimental effect on their health. Thirdly, it is important to highlight that numerous hospitals lack the presence of a qualified dietitian. This absence not only hinders access to essential nutritional guidance but also diminishes the patient’s ability to comprehend the dietary requirements they should adhere to. This underscores the critical need for a dietitian to be an integral part of the cardiac patient’s healthcare team, given the profound significance of proper nutrition in their treatment and recovery. The aforementioned analysis underscores the critical need for improvement in the domain of nutrition, as the current state of affairs impedes the comprehensive care of cardiac patients in hospitals. Recognizing that these diseases rely heavily on primary and secondary prevention measures is crucial. Compared to the recommendations, the primary issue regarding hospital nutrition lies in the reliance on meat-based dietary patterns rather than plant-based alternatives. Hospitals provide patients with products that are detrimental to their health, such as containing excessive amounts of salt, saturated fatty acids and sugar. The recommendations explicitly state that sweetened beverages should be avoided, including fruit juices as well as sugary carbonated and non-carbonated drinks. However, hospitals continue to offer these to patients. Furthermore, there is suboptimal inclusion of fiber and healthy fatty acids, and none of the hospitals have implemented the recommendation of consuming 30 g of unsalted nuts daily. In the majority of hospitals, fish was only included in one meal per week, which also fails to meet the recommended dietary guidelines. The reference diet developed during this study, based on the recommendations mentioned above, demonstrates that creating such meals does not require hard-to-find ingredients and is even more cost-effective than the dishes currently proposed in existing diets.

### 4.2. Strengths and Limitations

The positivity of the study lies in its pilot nature, paving the way for future research, a path that a dedicated research team is already ready to follow. The limited number of hospitals included in this study is a potential limitation, which may indicate a low level of representativeness. However, even with this small sample size, it highlights the magnitude of the problem when viewed from a certain perspective. Our calculations assume that approximately 146,425 patients per year did not receive exposure to an appropriate diet. As indicated by the data, inadequately nourished cardiac patients have a higher rate of hospital readmissions, leading to increased healthcare costs. Improper nutrition is often associated with prolonged hospital stays. Moreover, malnutrition can affect patients with both low and excessive body weight. One of the notable strengths of this study lies in its acknowledgment of the genuine challenges faced by cardiac patients within the Polish healthcare system. It sheds light on various aspects, starting from the lack of specialized wards catering to their specific needs to the provision of diets that are detrimental to their health and the absence of crucial nutrition education, which plays a vital role in the recovery process and could potentially alleviate the burden on the healthcare system. The study effectively demonstrates the reality of the meals that patients receive as part of their hospital diet therapy.

### 4.3. Agreement and Disagreement with Other Studies

The findings from various studies, including our own, provide evidence that the current nutrition provided to cardiac patients in hospitals is insufficient [15,16]. Lifestyle factors undoubtedly play a critical role in determining the occurrence and recurrence of cardiovascular events, with diet being the most extensively studied and substantiated component. It is noteworthy that diet holds equal significance in both primary and secondary prevention of CVD. The CORDIOPREV research has demonstrated that patients can effectively modify their dietary patterns when they receive adequate support from healthcare professionals and comprehensive nutritional education tailored to their cardiological condition [17]. One potential solution to address the nutrition problem among cardiac patients in the hospital is establishing an interdisciplinary team that focuses on the complexity of the issue [18]. Taking a comprehensive approach to nutrition is crucial, as interventions targeting individual nutrients alone, such as vitamin D or omega-3 fatty acids, have not shown sufficient effectiveness in reducing the incidence of CVDs [19]. Accumulating evidence suggests that incorporating targeted nutrition education for patients, emphasizing dietary modifications, has immense potential as a preventive strategy. This approach can decrease reliance on antihypertensive medications, optimize drug dosage and administration and enhance the overall effectiveness of pharmacological interventions. Moreover, this multifaceted approach can have far-reaching implications by positively impacting individuals’ cardiovascular risk profiles in both primary and secondary prevention of CVD [20]. Research confirms that patients are most receptive to lifestyle changes after a life-changing event, such as a myocardial infarction, and exposure to a well-planned diet is crucial [21,22]. However, one of the major challenges in nutritional CVD prevention is developing more effective strategies to inspire patients to change their diet and maintain these changes [13,22].

Multiple studies have shown that many hospitals serve fried foods, processed meats and sugar-sweetened beverages on a daily basis [16], which is also supported by our own data. A recent survey conducted in New York City hospitals revealed that none of the eight hospitals surveyed met all the required health standards. The study found that the diets provided to patients contained excessive calories from fat and saturated fat, inadequate fiber intake and sodium levels exceeding the recommended limits [23]. It is well established that a high sodium intake significantly increases the susceptibility to CVD [24]. For cardiac patients, gradually transitioning to less salty foods is advisable to encourage salt reduction in their ongoing management [20]. The hospital diet should serve as a model and an encouragement to change habits; unfortunately, the currently proposed diet in the surveyed Polish hospitals contains an excessively high amount of salt. Moreover, patients consuming food in healthcare settings expect the available options to be nutritious and in line with recommended guidelines [16].

The 2021 guidelines from the European Society of Cardiology emphasize the importance of proper nutrition in the treatment of cardiac patients. A healthy diet based on the Mediterranean diet has been shown to reduce the risk of CVDs and other chronic diseases [10]. The health benefits of this dietary approach have also been demonstrated in primary and secondary cardiovascular prevention [25]. Furthermore, research has shown that adherence to the Mediterranean diet not only provides significant health benefits but also leads to reduced hospitalization durations [26]. Therefore, implementing these dietary recommendations in hospital settings is of paramount importance. It should be emphasized that poor dietary quality has surpassed all other risk factors, contributing to approximately 11 million deaths and accounting for nearly 50% of global CVD mortality [27]. Consequently, addressing dietary quality becomes a critical step in reducing morbidity and mortality associated with CVDs.

In everyday clinical practice for chronic diseases, nutritional issues are often overlooked [9]. Compelling evidence has demonstrated the utmost significance of nutrition for patients perceiving that their meals have been meticulously crafted to meet their specific nutritional requirements. Dissatisfaction with hospital meals has been correlated with decreased consumption, which subsequently increases vulnerability to malnutrition and rehospitalization [15]. The role of diet and nutrition directly impacts cardiovascular health, which, in turn, has the potential to influence readmission rates for heart disease. This provides hospitals with an economic incentive to invest in conscientious dietary options [28]. There is also a new trend of incorporating an all-vegetarian menu into hospital patient populations, which has been shown in Florida hospitals to have positive health benefits for both patients and the hospital budget. These data demonstrate that offering patients a healthy and adequate diet does not increase healthcare costs but has rather positive consequences [28,29].

It is worth emphasizing that as early as 2018 [11], concerns were raised regarding the poor quality of nutrition in hospitals, and as our data show, no significant improvements have been made, with diets in many hospitals still being poorly planned. This indicates that the topic is not sufficiently publicized, and the importance of nutrition is not fully understood. It is crucial that the diet is properly tailored to the patient’s nutritional status and health, as being either underweight or obese has been associated with a longer stay [6]. Giving due importance to the issue of hospital nutrition and adhering to recommended guidelines are imperative.

A study has shown that implementing individualized nutritional support, as opposed to standard hospital food, significantly reduces the risk of mortality and major cardiovascular events. Therefore, emphasizing and implementing these recommendations can substantially impact patient outcomes [30].

## 5. Conclusions

This study shows a clear gap in the Polish healthcare system, wherein a substantial educational potential remains squandered given the considerable influx of patients requiring cardiological care admitted to Polish hospitals. Moreover, it highlights significant deficiencies in the nutritional care provided to cardiac patients in the Polish healthcare system. Hospitals continue to neglect recommended dietary plans, adversely affecting patient health. Many hospitals lack specialized cardiological diets, offering instead general diets that fail to meet patient needs. Nutrition education during hospital stays is inadequate, leading to potential errors in post-discharge diets. Improvements are needed in adopting plant-based diets, reducing processed meat, saturated fatty acids, sugar and salt and including fiber and healthy fatty acids. Implementing recommended guidelines, such as regular fish consumption, avoiding sweetened beverages and providing unsalted nuts, is crucial for better care and patient well-being. It is currently well established that a healthy diet is a cornerstone for CVD primary and secondary prevention. To effectively tackle these challenges, it is imperative to enact policy reforms, launch comprehensive educational campaigns and enhance the synergy among healthcare professionals. By acknowledging and proactively resolving the disparities highlighted in this research, we can enhance the management of cardiac patients and ultimately enhance their overall well-being. This pilot study not only sheds light on current issues but also lays the foundation for promising future research avenues.

## Figures and Tables

**Table 1:** Features of a healthy diet according to ESC guidelines [10].

The Predominance of Plant-Based Food Patterns and the Reduction in Animal-Based Ones
Limitation of saturated fatty acids to <10% of energy intake and their replacement by PUFAs, MUFAs and carbohydrates from whole grains
Minimalization of the amount of unsaturated trans fatty acids in the diet and exclusion of highly processed products
<5g total salt intake per day
30–45 g of fiber per day, optimally from whole grains
≥200 g of fruit per day (≥2–3 servings) ≥200 g of vegetables per day (≥2–3 servings)
Reduction in the consumption of red meat to the maximum 350–500 g per week, especially processed meat
Eating fish 1–2 times a week, especially fatty fish
Consuming 30 g of unsalted nuts per day
Limitation of alcohol consumption to a maximum of 100 g/week
Avoidance of sweetened beverages, including fruit juices and sweet carbonated and non-carbonated drinks

Abbreviations: ESC—European Society of Cardiology.

**Table 2:** The characteristics of the hospitals.

Variables	Hospital						
	1	2	3	4	5	6	7
Type of the hospital (degree of reference)	3rd	2nd	3rd	3rd	3rd	1st	3rd
Total number of beds	1035	452	683	677	363	176	321
Number of beds in internal medicine unit	386	96	21	87	45	52	50
Number of beds in cardiology unit	63	32	20	250	76	0	171
Presence of dietician	No	No	No	Yes	Yes	Yes	Yes
Nutrition education	Yes	Yes	No	Yes	Yes	Yes	Yes
The person responsible for nutrition education	Specialists outside the hospital (occasionally)	Doctor	-	Dietician, doctor, nurse	Dietician, doctor, nurse	Dietician, doctor, nurse	Dietician, doctor, nurse
Place of meal preparation	Catering	Catering	Kitchen in the hospital	Kitchen in the hospital	Catering	Kitchen in the hospital	Kitchen in the hospital

**Table 3:** Energy and carbohydrate content.

Hospital	M	SD	Mini	Max	*p*-Value **
Energy (kcal) (F = 12307.61; *p* < 0.001 *)					
Hospital 1	2102.07	225.04	1816.34	2558.38	0.764
Hospital 2	2358.78	69.31	2226.81	2448.24	0.001
Hospital 3	1966.56	96.46	1811.74	2087.37	0.997
Hospital 4	1783.75	57.43	1701.71	1881.67	0.078
Hospital 5	1930.80	94.88	1812.60	2155.87	0.914
Hospital 6	2214.67	200.95	1936.56	2610.27	0.073
Hospital 7	2147.87	193.29	1810.47	2448.25	0.376
Reference (standardized menu)	2005.36	20.64	1984.31	2039.82	-
Total amount of carbohydrates (F = 14789.56; *p* < 0.001 *)					
Hospital 1	305.31	23.42	266.56	335.13	0.152
Hospital 2	313.61	17.84	297.40	342.39	0.041
Hospital 3	296.36	19.22	262.96	320.93	0.606
Hospital 4	256.89	11.35	236.96	273.61	0.204
Hospital 5	295.79	15.72	267.12	319.26	0.644
Hospital 6	330.95	23.53	308.43	378.07	<0.001
Hospital 7	323.93	24.75	295.07	378.07	0.001
Reference (standardized menu)	281.09	11.37	266.26	298.17	-
Percentage of energy from carbohydrates (F = 16905.28; *p* < 0.001 *)					
Hospital 1	53.72	4.20	47.39	61.55	0.012
Hospital 2	48.44	2.18	45.86	51.66	1.000
Hospital 3	54.29	2.20	52.08	58.99	0.005
Hospital 4	51.73	2.24	47.29	53.92	0.243
Hospital 5	55.11	2.41	52.06	58.66	0.001
Hospital 6	56.08	4.86	46.41	63.33	<0.001
Hospital 7	56.55	3.61	52.18	62.12	<0.001
Reference (standardized menu)	47.91	1.89	45.21	50.17	-
Digestible carbohydrates (F = 13424.08; *p* < 0.001 *)					
Hospital 1	279.68	21.63	243.90	302.89	0.001
Hospital 2	284.35	17.34	268.19	314.86	0.001
Hospital 3	265.74	17.78	236.36	283.61	0.058
Hospital 4	229.35	10.92	210.00	240.88	0.946
Hospital 5	264.23	14.21	237.12	283.48	0.083
Hospital 6	307.76	23.65	286.53	355.29	<0.001
Hospital 7	301.46	23.18	280.08	355.29	<0.001
Reference	238.42	10.78	224.78	253.90	-
Fiber (F = 5870.27; *p* < 0.001 *)					
Hospital 1	25.64	3.49	19.60	32.23	<0.001
Hospital 2	29.26	3.11	25.78	34.70	<0.001
Hospital 3	30.61	3.73	26.59	38.50	<0.001
Hospital 4	27.54	3.08	24.04	32.72	<0.001
Hospital 5	31.56	2.75	25.95	35.78	<0.001
Hospital 6	23.20	2.15	19.40	27.16	<0.001
Hospital 7	22.47	3.11	14.99	27.16	<0.001
Reference (standardized menu)	42.68	2.55	39.39	45.99	-

M—mean, SD—standard deviation, * one-way ANOVA, ** post hoc Dunnett’s two-tailed test (multiple comparisons to a control are also referred to as many-to-one comparisons).

**Table 4:** Protein content.

Hospital	M	SD	Mini	Max	*p*-Value **
Total amount of protein (F = 7647.82; *p* < 0.001 *)					
Hospital 1	73.95	9.93	59.16	92.37	<0.001
Hospital 2	99.05	7.06	88.81	110.67	0.737
Hospital 3	76.19	8.58	60.14	89.47	<0.001
Hospital 4	79.58	9.24	65.39	94.59	<0.001
Hospital 5	80.93	6.33	72.44	89.17	<0.001
Hospital 6	83.14	5.97	74.43	92.17	<0.001
Hospital 7	82.07	8.00	64.63	92.17	<0.001
Reference (standardized menu)	104.63	5.42	99.66	110.50	-
Percentage of energy from protein (F = 6927.64; *p* < 0.001 *)					
Hospital 1	14.14	1.65	11.04	16.39	<0.001
Hospital 2	16.74	0.84	15.02	17.72	<0.001
Hospital 3	15.64	1.69	12.88	18.35	<0.001
Hospital 4	17.62	1.55	15.21	19.44	0.003
Hospital 5	16.95	1.21	15.51	18.90	<0.001
Hospital 6	15.20	2.10	11.83	19.19	<0.001
Hospital 7	15.40	1.86	12.34	19.19	<0.001
Reference (standardized menu)	21.06	1.05	20.04	22.46	-
Plant-based protein (F = 5740.80; *p* < 0.001 *)					
Hospital 1	31.69	5.06	25.88	40.58	<0.001
Hospital 2	34.04	4.38	29.80	42.46	<0.001
Hospital 3	32.83	5.31	25.94	42.07	<0.001
Hospital 4	35.50	3.95	31.85	43.69	<0.001
Hospital 5	36.46	1.96	33.84	40.67	<0.001
Hospital 6	34.04	2.51	30.74	40.06	<0.001
Hospital 7	33.86	2.58	30.74	40.06	<0.001
Reference (standardized menu)	55.79	4.69	48.32	60.81	-
Animal-based protein (F = 2150.84; *p* < 0.001 *)					
Hospital 1	42.00	10.52	30.00	59.57	0.659
Hospital 2	64.75	8.67	52.26	79.11	0.007
Hospital 3	43.16	8.19	28.66	54.04	0.831
Hospital 4	43.84	10.18	31.72	61.10	0.932
Hospital 5	44.22	6.43	35.25	50.96	0.941
Hospital 6	48.93	5.71	41.53	58.15	1.000
Hospital 7	48.03	7.39	32.78	58.15	1.000
Reference (standardized menu)	48.04	9.86	38.28	62.00	-

M—mean, SD—standard deviation, * one-way ANOVA, ** post hoc Dunnett’s two-tailed test (multiple comparisons to a control are also referred to as many-to-one comparisons).

**Table 5:** Fatty acid content.

Hospital	M	SD	Mini	Max	*p*-Value **
Total amount of fat (F = 1697.67; *p* < 0.001 *)					
Hospital 1	70.61	17.99	52.48	113.35	0.601
Hospital 2	85.13	6.03	76.65	91.61	0.011
Hospital 3	59.77	5.40	53.47	70.09	1.000
Hospital 4	55.01	4.66	47.42	60.16	0.953
Hospital 5	53.97	5.69	47.26	65.92	0.869
Hospital 6	67.20	20.62	39.60	114.07	0.913
Hospital 7	63.20	14.57	39.60	81.07	1.000
Reference (standardized menu)	60.90	6.08	54.62	69.48	-
Percentage of energy from fat (F = 3635.99; *p* < 0.001 *)					
Hospital 1	29.80	4.30	24.03	39.67	0.608
Hospital 2	32.47	1.94	29.53	34.83	0.076
Hospital 3	27.12	2.21	24.44	31.17	1.000
Hospital 4	27.73	2.39	24.70	31.26	1.000
Hospital 5	24.83	1.76	22.49	27.19	0.840
Hospital 6	26.74	6.09	18.16	40.03	1.000
Hospital 7	26.07	4.44	18.16	32.37	0.998
Reference (standardized menu)	26.97	2.77	23.81	30.82	-
Saturated fatty acids (F = 2027.01; *p* < 0.001 *)					
Hospital 1	29.24	4.21	23.59	38.38	<0.001
Hospital 2	32.83	3.76	27.55	36.96	<0.001
Hospital 3	27.85	2.92	23.41	33.35	<0.001
Hospital 4	18.66	2.09	15.44	20.88	0.542
Hospital 5	24.20	2.63	20.98	29.12	0.003
Hospital 6	32.84	7.66	21.16	46.71	<0.001
Hospital 7	30.26	6.51	21.16	42.16	<0.001
Reference (standardized menu)	14.56	2.86	11.51	18.65	-
Polyunsaturated fatty acids (F = 1000.07; *p* < 0.001 *)					
Hospital 1	9.91	4.27	4.09	20.09	<0.001
Hospital 2	12.10	2.04	9.97	15.94	<0.001
Hospital 3	7.87	2.06	4.79	10.77	<0.001
Hospital 4	12.71	1.27	10.51	14.32	<0.001
Hospital 5	7.47	1.67	5.41	10.15	<0.001
Hospital 6	7.34	3.12	4.34	14.81	<0.001
Hospital 7	7.25	3.02	4.08	14.35	<0.001
Reference (standardized menu)	20.92	2.33	18.22	24.56	-
*n*-3 acids (F = 328.40; *p* < 0.001 *)					
Hospital 1	2.31	1.39	0.65	5.64	0.979
Hospital 2	2.95	0.84	2.25	4.61	0.987
Hospital 3	1.60	0.75	0.59	2.74	0.169
Hospital 4	1.90	0.41	1.26	2.37	0.575
Hospital 5	1.20	0.48	0.67	1.87	0.022
Hospital 6	1.51	0.95	0.65	3.83	0.111
Hospital 7	1.48	0.94	0.53	3.71	0.098
Reference (standardized menu)	2.63	0.22	2.42	2.94	-
*n*-6 acids (F = 1251.11; *p* < 0.001 *)					
Hospital 1	7.59	3.02	3.45	14.41	<0.001
Hospital 2	9.14	1.35	7.56	11.31	<0.001
Hospital 3	6.26	1.68	3.85	8.41	<0.001
Hospital 4	10.81	0.90	9.24	11.94	<0.001
Hospital 5	6.27	1.26	4.65	8.27	<0.001
Hospital 6	5.82	2.18	3.68	10.96	<0.001
Hospital 7	5.76	2.10	3.55	10.61	<0.001
Reference (standardized menu)	18.28	2.35	15.28	21.77	-

M—mean, SD—standard deviation, * one-way ANOVA, ** post hoc Dunnett’s two-tailed test (multiple comparisons to a control are also referred to as many-to-one comparisons).

**Table 6:** Salt and sodium content.

Hospital	M	SD	Mini	Max	*p*-Value **
Sodium (F = 3411.58; *p* < 0.001 *)					
Hospital 1	4775.18	959.62	2915.91	5998.96	0.768
Hospital 2	4607.82	637.18	3644.99	5297.30	0.984
Hospital 3	4431.87	730.49	3112.99	5729.69	1.000
Hospital 4	4938.30	542.37	3941.12	5492.84	0.508
Hospital 5	4479.40	698.99	3009.38	5470.33	1.000
Hospital 6	4619.53	377.62	3797.37	5069.05	0.971
Hospital 7	4601.40	404.12	3678.83	5065.45	0.981
Reference (standardized menu)	4367.64	194.53	4218.51	4591.51	-
Salt (F = 3416.12; *p* < 0.001 *)					
Hospital 1	11.94	2.40	7.30	15.00	0.768
Hospital 2	11.53	1.59	9.12	13.25	0.984
Hospital 3	11.09	1.83	7.79	14.33	1.000
Hospital 4	12.35	1.36	9.86	13.74	0.509
Hospital 5	11.20	1.75	7.53	13.68	1.000
Hospital 6	11.56	0.94	9.50	12.68	0.971
Hospital 7	11.51	1.01	9.20	12.67	0.981
Reference (standardized menu)	10.93	0.49	10.55	11.49	-

M—mean, SD—standard deviation, * one-way ANOVA, ** post hoc Dunnett’s two-tailed test (multiple comparisons to a control are also referred to as many-to-one comparisons).

**Table 7:** Products divided into subgroups.

Hospital	M	SD	Mini	Max	*p*-Value **
Fruits (F = 723.54; *p* < 0.001 *)					
Hospital 1	222.13	82.60	138.75	334.95	0.022
Hospital 2	302.08	38.68	228.75	341.25	0.998
Hospital 3	181.06	58.06	138.75	301.23	<0.001
Hospital 4	123.16	68.55	36.43	178.10	<0.001
Hospital 5	159.66	79.48	111.00	336.00	<0.001
Hospital 6	159.40	39.92	138.75	261.25	<0.001
Hospital 7	140.73	13.00	122.50	174.75	<0.001
Reference (standardized menu)	316.93	19.07	300.00	346.20	-
Vegetables (F = 976.51; *p* < 0.001 *)					
Hospital 1	405.29	122.86	188.75	578.40	<0.001
Hospital 2	618.17	103.37	492.24	767.15	0.198
Hospital 3	505.80	174.03	290.73	897.74	0.001
Hospital 4	390.16	95.71	265.26	490.29	<0.001
Hospital 5	344.32	82.91	209.17	438.37	<0.001
Hospital 6	288.80	92.30	73.18	393.36	<0.001
Hospital 7	280.55	95.49	73.18	393.36	<0.001
Reference (standardized menu)	759.97	132.29	574.61	861.45	-
Whole-grain products (F = 65.67; *p* < 0.001 *)					
Hospital 1	24.00	40.61	0.00	100.00	0.005
Hospital 2	42.00	45.50	0.00	110.00	0.095
Hospital 3	47.50	34.92	0.00	80.00	0.012
Hospital 4	101.00	48.64	0.00	130.00	0.804
Hospital 5	135.00	129.07	0.00	260.00	1.000
Hospital 6	15.00	24.15	0.00	50.00	0.002
Hospital 7	15.00	24.15	0.00	50.00	0.002
Reference (standardized menu)	135.00	69.76	50.00	200.00	-
Legumes					
Hospital 1	28.99	12.04	20.48	37.50	
Hospital 2	41.25	-	41.25	41.25	
Hospital 3	25.42	4.69	20.00	28.13	
Hospital 4	62.50	-	62.50	62.50	
Hospital 5	-	-	-	-	
Hospital 6	43.75	-	43.75	43.75	
Hospital 7	43.75	-	43.75	43.75	
Reference (standardized menu)	43.25	11.17	26.25	52.50	
Nuts and beans					
Hospital 1	10.27	-	10.27	10.27	
Hospital 2	-	-	-	-	
Hospital 3	7.50	3.54	5.00	10.00	
Hospital 4	11.90	-	11.90	11.90	
Hospital 5	-	-	-	-	
Hospital 6	-	-	-	-	
Hospital 7	-	-	-	-	
Reference (standardized menu)	30.00	0.00	30.00	30.00	
Fish					
Hospital 1	61.08	23.81	40.00	83.33	-
Hospital 2	233.33	-	-	-	-
Hospital 3	58.81	42.48	9.76	83.33	-
Hospital 4	153.33	-	-	-	-
Hospital 5	120.00	-	-	-	-
Hospital 6	88.24	-	-	-	-
Hospital 7	88.24	-	-	-	-
Reference (standardized menu)	216.00	-	-	-	-
Red meat					
Hospital 1	53.68	31.03	25.67	102.27	
Hospital 2	89.11	49.25	50.53	159.90	
Hospital 3	84.34	41.99	32.08	131.20	
Hospital 4	175.36	23.20	158.95	191.76	
Hospital 5	72.78	40.32	32.08	127.92	
Hospital 6	79.90	23.40	36.90	98.18	
Hospital 7	79.90	23.40	36.90	98.18	
Reference (standardized menu)	-	-	-	-	
Processed meat (F = 717.13; *p* < 0.001 *)					
Hospital 1	36.63	11.43	17.70	46.08	0.001
Hospital 2	47.23	12.14	29.50	57.82	0.053
Hospital 3	39.39	5.78	33.98	46.08	0.002
Hospital 4	37.29	10.24	22.66	46.26	0.003
Hospital 5	44.63	17.39	28.32	76.80	0.015
Hospital 6	33.48	10.45	22.66	47.20	<0.001
Hospital 7	33.48	10.45	22.66	47.20	<0.001
Reference (standardized menu)	67.20	19.20	38.40	76.80	-
Sugar (F = 1327.33; *p* < 0.001 *)					
Hospital 1	54.46	12.79	41.25	83.95	<0.001
Hospital 2	52.46	3.61	48.13	56.57	<0.001
Hospital 3	49.02	5.66	44.89	60.09	<0.001
Hospital 4	2.07	1.45	0.40	3.75	1.000
Hospital 5	42.98	6.12	35.20	56.10	<0.001
Hospital 6	49.43	3.41	46.25	56.25	<0.001
Hospital 7	45.80	9.38	20.00	54.25	<0.001
Reference (standardized menu)	3.09	1.28	1.88	4.43	-

M—mean, SD—standard deviation, * one-way ANOVA, ** post hoc Dunnett’s two-tailed test (multiple comparisons to a control are also referred to as many-to-one comparisons).

**Table 8:** The summary of the ESC criteria met by hospitals [10].

Recommendation	Hospital						
	1	2	3	4	5	6	7
The predominance of plant-based and reduction in animal-based food pattern	−	−	−	−	−	−	−
Saturated fatty acids should account for <10% of energy intake	−	−	−	+	−	−	−
<5 g total salt intake per day	−	−	−	−	−	−	−
30–45 g of fiber per day	−	−	+	−	+	−	−
≥200 g of fruit per day (≥2–3 servings)	−	+	−	−	−	−	−
≥200 g of vegetables per day (≥2–3 servings)	+	+	+	+	+	+	+
Reduction In the consumption of red meat to the maximum 350–500 g per week, especially processed meat	+	+	+	+	+	+	+
Eat fish 1–2 times a week	+	+	+	+	+	+	+
30 g of unsalted nuts per day	−	−	−	−	−	−	−
Alcohol consumption should be limited to a maximum of 100 g/week	+	+	+	+	+	+	+
Sweetened beverages, including fruit juices and sweet carbonated drinks and non-carbonated, should be avoided	−	−	−	+	−	−	−

Red colour—a recommendation not fulfiled; Green colour—a recommendation fulfiled.

## Data Availability

The raw data supporting the conclusions of this article will be made available by the authors, without undue reservation.

## Supplementary Materials

The following supporting information can be downloaded at https://www.mdpi.com/article/10.3390/nu15214606/s1, Supplementary S1: The questionnaire, Supplementary S2: Reference diet.
