# Supplementary material for: Examining the Nutrition of Cardiological Patients in Hospitals: Evaluating the Discrepancy between Received Diets and Reference Diet Based on ESC 2021 Guidelines—Hospital Diet Medical Investigation) (HDMI) Study
Source: Nutrients. 2023 Oct 30;15(21):4606. doi: 10.3390/nu15214606 (PMC10650773; doi:10.3390/nu15214606)
Supplement: Supplementary file 1 [file nutrients-15-04606-s001.zip › nutrients-2681502-supplementary.pdf]

Supplementary S1: The questionnaire

Evaluation of patient nutrition in internal medicine and cardiology ward – Cardiology and Internal Medicine Departments Diet (CIDDA assessment)

Dear Sir or Madam,

as part of the activities of the Student Scientific Society of Lifestyle Medicine operating at III Department of Internal Diseases and Cardiology of the Medical University of Warsaw, we run study with the acronym CIDDA (Cardiology and Internal Medicine Departments Diet Assessment), in which we analyse menus of hospital diets received by patients in wards: cardiology and internal medicine.

The aim of our scientific investigations is to answer the question: is the composition of the diets proposed in Polish cardiology and internal medicine departments in line with the latest ESC guidelines 2021 on cardiovascular prevention. We want to perform a multicenter research. Its results will show the current situation of nutrition in Polish hospitals and create an opportunity to discuss the scale of the problem and any necessary changes. Please complete the questionnaire below and attach the decade menu at the end of the files (from 10 consecutive days) or, in the absence of it, weekly (7-day) basic (standard) diet and for a cardiological patient, if they are available in your ward. It is best that the menus contain the grammage of the ingredients. Any data you provide to us will be anonymized so that they do not indicate to a specific facility. They will be used only for this study. We will be very grateful for your help, without which the study will not be possible.

If you have any doubts about the survey, menus or the study itself, please contact us e-mail: badanie.kardiologiczne@gmail.com

Gratefully,

Alicja Jodczyk

Dr. med. Alicja Kucharska

Dr. med. Daniel Śliż - supervisor of SKN Lifestyle Medicine of the Medical University of Warsaw

1. Please provide an e-mail address
2. Name and Surname
3. E- mail address for contact
4. Name of the hospital
5. Type of the hospital (choose only one answer)
- 1st degree of reference
- 2nd degree of reference
- 3rd degree of reference
6. City
7. How many beds in total are there in your hospital?
8. How many internal medicine beds are there in your hospital? *
9. How many cardiology beds are there in your hospital? *
10. Is there a dietitian in your ward? (choose one answer)
- Yes
- No
11. Is there nutrition education for patients in your ward? (you can choose more then one answer)
- Yes, by a nutritionist
- Yes, by a doctor
- Yes, by a nurse

- Yes, occasionally by people outside the hospital
- NO

12. Where are meals prepared for patients in your hospital? *(choose one answer)

- Hospital kitchen
- Catering

13. Please provide the contact to the hospital kitchen or the person responsible for nutrition in your hospital (in the absence of a kitchen and the use of catering hospital, please enter "not applicable")
14. Name and contact of the catering company (if you do not use hospital from the services of a catering company, please enter "not applicable")
15. Is a diabetic patient on a special diet in your hospital?
16. Does a "cardiac" patient in your hospital get a different dedicated diet?
17. Can a patient choose a vegetarian (meatless) diet in your hospital? *
18. Can a patient choose a vegetarian (meatless) diet in your hospital? *

Please attach the menus below or send them by e-mail: badanie.kardiologiczne@gmail.com

Supplementary S2: Reference diet

Menu for: METABOLIC MODEL DIET - 52,93 years per day 24.11.2022

Meal time		quantity	product/dish
Breakfast	8:0	150,00	Frozen strawberries [g]
Breakfast	8:0	30,00	Sunflower, shelled seeds [g]
Breakfast	8:0	100,00	Cucumber [g]
Breakfast	8:0	10,00	Margarine "Rama" [g]
Breakfast	8:0	100,00	Bread Graham [g]
Breakfast	8:0	250,00	Tea, infusion without sugar [ml, g]
Breakfast	8:0	100,00	Turkey ham [g]
Breakfast	8:0	330,00	Oatmeal on milk 2% [g]
Dinner	12:0	330,00	Barley soup with millet groats [g]
Dinner	12:0	250,00	Chicken with curry and vegetables [g]
Dinner	12:0	180,00	Cooked brown rice [g]
Dinner	12:0	150,00	White cabbage and carrot salad [g]
Dinner	12:0	10,00	Universal rapeseed oil [g]
Dinner	12:0	150,00	Apple [g]
Supper	17:0	30,00	Natural yoghurt 2% fat [g]
Supper	17:0	10,00	Chives [g]
Supper	17:0	70,00	Curd [g]
Supper	17:0	100,00	Bread Graham [g]
Supper	17:0	150,00	Cabbage salad, red beans, cucumbers [g]
Supper	17:0	70,00	Beans, cooked [g]
Supper	17:0	5,00	Universal rapeseed oil [g]
Supper	17:0	250,00	Tea, infusion without sugar [ml, g]

Summary of the nutritional values of the menu:

Nutrient	of 24 hours	Breakfast	Lunch	Dinner	Tea	Supper
Energy [kcal]	1984,3104	797,8468	0,0000	691,2975	0,0000	495,1661
Energy % from a meal (without eating)		40,21	0,00	34,84	0,00	24,95
Total protein [g]	110,4977	43,0897	0,0000	36,7609	0,0000	30,6471
Fat [g]	55,7349	29,5066	0,0000	14,9574	0,0000	11,2709
Total carbohydrates [g]	281,3572	96,9597	0,0000	110,2033	0,0000	74,1942
Isoleucine [mg]	5312,3586	2016,9257	0,0000	1862,8821	0,0000	1432,5508
Leucine [mg]	8251,1545	3132,6664	0,0000	2711,7458	0,0000	2406,7423
Lysine [mg]	7516,6556	2978,5013	0,0000	2786,4906	0,0000	1751,6637
Methionine [mg]	2506,5365	973,5161	0,0000	925,0110	0,0000	608,0094
Cystine [mg]	1547,6517	642,2272	0,0000	502,1386	0,0000	403,2859
Phenylalanine [mg]	4718,3956	1801,6983	0,0000	1474,8863	0,0000	1441,8110
Tryptophan [mg]	1479,7275	538,0449	0,0000	569,8965	0,0000	371,7861
Sodium [mg]	4569,1529	1153,0665	0,0000	2713,5918	0,0000	702,4946
Potassium [mg]	4077,8086	1507,4887	0,0000	1682,2484	0,0000	888,0715
Calcium [mg]	801,5204	355,2542	0,0000	199,4899	0,0000	246,7763
Phosphorus [mg]	2094,6773	940,8129	0,0000	610,8920	0,0000	542,9724
Magnesium [mg]	618,6661	282,0457	0,0000	195,4627	0,0000	141,1577
Iron [mg]	15,6058	6,7007	0,0000	3,8874	0,0000	5,0177
Zinc [mg]	13,5765	6,0671	0,0000	3,3462	0,0000	4,1632
Copper [mg]	2,2135	1,2553	0,0000	0,5252	0,0000	0,4330
Mangan [mg]	10,0956	4,6059	0,0000	2,4819	0,0000	3,0078
Retinol [µg]	182,1295	138,9120	0,0000	15,2175	0,0000	28,0000
Vitamin B1 (thiamine) [mg]	1,8846	0,9033	0,0000	0,5490	0,0000	0,4323
Vitamin B2 (riboflavin) [mg]	1,8754	0,8235	0,0000	0,4761	0,0000	0,5758
Niacyna [mg]	34,3128	13,3284	0,0000	15,6980	0,0000	5,2864
Vitamin C [mg]	177,8791	96,8400	0,0000	56,9641	0,0000	24,0750
Vitamin A [micro g]	1599,2197	166,7400	0,0000	1250,8378	0,0000	181,6419
Vitamin E [mg]	19,2878	10,9760	0,0000	5,7200	0,0000	2,5918
Vitamin B6 [mg]	2,8824	0,9480	0,0000	1,3727	0,0000	0,5617
Vitamin B12 [µg]	2,8001	1,5684	0,0000	0,5217	0,0000	0,7100
vitamin D [micro g]	0,9461	0,8644	0,0000	0,0097	0,0000	0,0720
Glucose [g]	10,6190	4,1040	0,0000	5,7266	0,0000	0,7884
Saccharose [g]	11,1297	3,0750	0,0000	6,4777	0,0000	1,5770

Fructose [g]	13,8372	4,1040	0,0000	9,0290	0,0000	0,7042
Lactose [g]	13,9141	8,4154	0,0000	2,2987	0,0000	3,2000
Starch [g]	182,8079	62,3336	0,0000	68,1359	0,0000	52,3384
Dietary fibre [g]	42,2682	13,1044	0,0000	15,9137	0,0000	13,2501
Folin [g]	454,0492	144,7778	0,0000	127,8499	0,0000	181,4215
Cholesterol [mg]	144,8882	69,9360	0,0000	60,6522	0,0000	14,3000
Fatty acids: total saturated [g]	12,1563	6,9861	0,0000	2,0886	0,0000	3,0816
Fatty acids: total monounsaturated [g]	20,1574	7,9821	0,0000	7,3543	0,0000	4,8210
Fatty acids: 18:2 [g]	17,5311	12,4982	0,0000	2,9760	0,0000	2,0569
Fatty acids: 18:3 [g]	2,3932	0,6282	0,0000	0,9788	0,0000	0,7862
Fatty acids: total polyunsaturated [g]	20,0022	13,1555	0,0000	4,0001	0,0000	2,8466
Acids n-3 [g]	2,4228	0,6282	0,0000	1,0084	0,0000	0,7862
Acids n-6 [g]	17,5694	12,5270	0,0000	2,9855	0,0000	2,0569
Salt [g]	11,4304	2,8863	0,0000	6,7860	0,0000	1,7581
Water [ml]	2277,8261	859,7590	0,0000	855,4440		0,0000
562,6231						
% of energy from protein	22,47					
% of energy from fat	24,93					
% of energy from carbohydrates assimilable.	48,55					
% energy from fibre	4,05					
	52,61					

Menu for: METABOLIC MODEL DIET - 52,93 years per day 25.11.2022

Meal	Hour	Quantity	Product/dish
Breakfast	8:0	50,00	Barley flakes [g]
Breakfast	8:0	50,00	Boiled eggs [g]
Breakfast	8:0	10,00	Margarine "Rama" [g]
Breakfast	8:0	100,00	Red pepper [g]
Breakfast	8:0	20,00	Lettuce [g]
Breakfast	8:0	10,00	Walnuts [g]
Breakfast	8:0	20,00	Pumpkin, seeds, shelled [g]
Breakfast	8:0	150,00	Apple [g]
Breakfast	8:0	50,00	Chicken ham [g]
Breakfast	8:0	200,00	Natural yoghurt 2% fat [g]
Breakfast	8:0	100,00	Bread Graham [g]
Dinner	12:0	140,00	Tomato [g]
Dinner	12:0	330,00	Ukrainian borscht with green beans [g]
Dinner	12:0	250,00	Potatoes (average) cooked [g]
Dinner	12:0	150,00	Chinese cabbage, pepper and apple salad [g]
Dinner	12:0	15,00	Universal rapeseed oil [g]
Dinner	12:0	150,00	Tangerines [g]
Dinner	12:0	250,00	Tea, infusion without sugar [ml, g]
Dinner	12:0	200,00	Steamed cod (fillet without skin) [g]
Supper	17:0	100,00	Bread Graham [g]
Supper	17:0	120,00	Lentil paste - without eggs (pate) [g]
Supper	17:0	100,00	Tomato [g]
Supper	17:0	10,00	Chives [g]

Summary of the nutritional values of the menu:

Nutrient	of 24 hours	Breakfast	Lunch	Dinner	Tea	Supper
Energy [kcal]	2039,8226	943,5668	0,0000	677,8683	0,0000	418,3875
Energy % from a meal (excluding snacking)		46,26	0,00	33,23	0,00	20,51
Total protein [g]	110,4925	42,8663	0,0000	46,0786	0,0000	21,5476
Fat [g]	54,6164	34,0326	0,0000	17,1494	0,0000	3,4344
Carbohydrates [g]	298,1728	124,7005	0,0000	91,6859	0,0000	81,7864
Isoleucine [mg]	5077,9723	2080,5170	0,0000	2074,4964	0,0000	922,9589
Leucine [mg]	8219,6078	3281,5730	0,0000	3368,4143	0,0000	1569,6205
Lysin [mg]	7746,7404	2552,1580	0,0000	4047,7190	0,0000	1146,8634
Methionine [mg]	2406,2172	989,3320	0,0000	1132,8136	0,0000	284,0716
Cystine [mg]	1628,9855	726,8220	0,0000	537,1669	0,0000	364,9966
Phenylalanine [mg]	4850,7792	1930,3990	0,0000	1851,9498	0,0000	1068,4304
Tryptophan [mg]	1355,4775	559,0940	0,0000	574,9354	0,0000	221,4481
Sodium [mg]	4218,5579	1337,4150	0,0000	2245,5304	0,0000	635,6125
Potassium [mg]	5908,9405	1603,9690	0,0000	3304,4428	0,0000	1000,5287
Calcium [mg]	706,2822	436,8500	0,0000	196,0805	0,0000	73,3517
Phosphorus [mg]	2010,1394	1068,0290	0,0000	583,4392	0,0000	358,6712
Magnesium [mg]	613,4080	287,6340	0,0000	214,6778	0,0000	111,0962
Iron [mg]	21,8145	9,6440	0,0000	6,4836	0,0000	5,6869
Zinc [mg]	14,7235	7,2434	0,0000	3,5756	0,0000	3,9045
Copper [mg]	2,3517	0,9147	0,0000	0,7480	0,0000	0,6890
Mangan [mg]	6,8346	2,4934	0,0000	2,1060	0,0000	2,2352
Retinol [µg]	244,0000	231,0400	0,0000	12,9600	0,0000	0,0000
Vitamin B1 (thiamine) [mg]	1,8553	0,5611	0,0000	0,6422	0,0000	0,6520
Vitamin B2 (riboflavin) [mg]	1,8712	1,0562	0,0000	0,5175	0,0000	0,2975
Niacyna [mg]	26,4235	8,8347	0,0000	11,6200	0,0000	5,9688
Vitamin C [mg]	316,5894	135,2826	0,0000	149,3793	0,0000	31,9275
Vitamin A [micro g]	1714,5670	721,5213	0,0000	653,7571	0,0000	339,2886
Vitamin E [mg]	21,6220	10,7173	0,0000	8,3891	0,0000	2,5156
Vitamin B6 [mg]	3,6668	1,0983	0,0000	2,0077	0,0000	0,5608
Vitamin B12 [µg]	3,4811	1,7531	0,0000	1,7280	0,0000	0,0000
vitamin D [micro g]	3,5319	1,5879	0,0000	1,9440	0,0000	0,0000
Glucose [g]	12,5325	4,0386	0,0000	6,8964	0,0000	1,5975

Saccharose [g]	24,2077	5,7225	0,0000	16,7530	0,0000	1,7322
Fructose [g]	17,9287	8,0285	0,0000	8,0523	0,0000	1,8479
Lactose [g]	6,4400	6,4400	0,0000	0,0000	0,0000	0,0000
Starch [g]	178,4728	72,9655	0,0000	44,0763	0,0000	61,4310
Dietary fibre [g]	44,2744	16,6053	0,0000	14,6729	0,0000	12,9962
Folin [g]	556,7629	164,6123	0,0000	280,7750	0,0000	111,3756
Cholesterol [mg]	305,3200	208,1200	0,0000	97,2000	0,0000	0,0000
Fatty acids: total saturated [g]	11,5089	9,5025	0,0000	1,3926	0,0000	0,6138
Fatty acids: total monounsaturated [g]	20,4798	10,2425	0,0000	9,6030	0,0000	0,6343
Fatty acids: 18:2 [g]	15,2281	10,3985	0,0000	3,1868	0,0000	1,6428
Fatty acids: 18:3 [g]	2,6465	0,9679	0,0000	1,4290	0,0000	0,2496
Fatty acids: total polyunsaturated [g]	18,2246	11,4721	0,0000	4,8602	0,0000	1,8923
Acids n-3 [g]	2,9383	1,0459	0,0000	1,6428	0,0000	0,2496
Acids n-6 [g]	15,2754	10,4264	0,0000	3,2062	0,0000	1,6428
Salt [g]	10,5518	3,3447	0,0000	5,6182	0,0000	1,5889
Water [ml]	1967,1607	481,3668	0,0000	1271,8268	0,0000	213,9671
% of energy from protein	21,89					
% of energy from fat	23,81					
% of energy from carbohydrates digestible.	50,17					
% energy from fibre	4,13					
	54,30					

Menu for: METABOLIC MODEL DIET - 52,94 years per day 26.11.2022

Meal	Hour	Quantity	Product/dish
Breakfast	8:0	50,00	Oatmeal [g]
Breakfast	8:0	30,00	Pumpkin, seeds, shelled [g]
Breakfast	8:0	150,00	Plums [g]
Breakfast	8:0	120,00	Soya paste [g]
Breakfast	8:0	100,00	Cucumber [g]
Breakfast	8:0	250,00	Tea, sugar-free [ml, g]
Breakfast	8:0	20,00	Butter lettuce [g]
Breakfast	8:0	100,00	Bread Graham [g]
Breakfast	8:0	200,00	Natural yoghurt 2% fat [g]
Dinner	12:0	150,00	Cooked buckwheat groats [g]
Dinner	12:0	330,00	Vegetable soup [g]
Dinner	12:0	250,00	Chicken cooked in vegetables [g]
Dinner	12:0	150,00	Salad of white cabbage, carrots, tomatoes, peppers [g]
Dinner	12:0	10,00	Universal rapeseed oil [g]
Dinner	12:0	150,00	Apple [g]
Dinner	12:0	250,00	Tea, Sugar-free [ml, g]
Supper	17:0	10,00	Chives [g]
Supper	17:0	30,00	Natural yoghurt 2% fat [g]
Supper	17:0	60,00	Ser twarogowy półtłusty [g]
Supper	17:0	10,00	Chives [g]
Supper	17:0	10,00	Margarine "Rama" [g]
Supper	17:0	150,00	Radish [g]
Supper	17:0	250,00	Tea, infusion without sugar [ml, g]
Supper	17:0	100,00	Bread Graham [g]
Supper	17:0	20,00	Butter lettuce [g]

Summary of the nutritional values of the menu:

Nutrient	of 24 hours	Breakfast	Lunch	Dinner	Tea	Supper
Energy [kcal]	1999,5055	944,7439	0,0000	638,1348	0,0000	416,6268
Energy % from a meal (without eating)		47,25	0,00	31,91	0,00	20,84
Total protein [g]	100,4700	45,3928	0,0000	32,1326	0,0000	22,9446
Fat [g]	63,7313	29,9035	0,0000	21,3438	0,0000	12,4840
Total carbohydrates [g]	278,8987	133,3554	0,0000	87,4877	0,0000	58,0556
Isoleucine [mg]	4803,5483	2146,3870	0,0000	1602,3633	0,0000	1054,7980
Leucine [mg]	7492,5419	3462,5234	0,0000	2265,3065	0,0000	1764,7120
Lysin [mg]	6077,4850	2565,3777	0,0000	2354,5363	0,0000	1157,5710
Methionine [mg]	2092,9353	862,4979	0,0000	731,3164	0,0000	499,1210
Cystine [mg]	1663,8796	839,0252	0,0000	489,1334	0,0000	335,7210
Phenylalanine [mg]	4310,4257	2061,9432	0,0000	1199,7995	0,0000	1048,6830
Tryptophan [mg]	1391,9051	631,9850	0,0000	487,9371	0,0000	271,9830
Sodium [mg]	4240,4551	957,5499	0,0000	2752,4142	0,0000	530,4910
Potassium [mg]	4813,8743	2310,2368	0,0000	1816,7375	0,0000	686,9000
Calcium [mg]	987,6306	557,2888	0,0000	238,0908	0,0000	192,2510
Phosphorus [mg]	2297,9881	1315,5838	0,0000	592,7093	0,0000	389,6950
Magnesium [mg]	755,0797	434,6710	0,0000	229,7417	0,0000	90,6670
Iron [mg]	23,2695	13,8824	0,0000	5,6162	0,0000	3,7709
Zinc [mg]	17,0238	8,8093	0,0000	5,0834	0,0000	3,1311
Copper [mg]	2,7288	1,8277	0,0000	0,6175	0,0000	0,2836
Mangan [mg]	11,6601	6,1770	0,0000	2,9951	0,0000	2,4880
Retinol [µg]	155,3345	28,0000	0,0000	12,7345	0,0000	114,6000
Vitamin B1 (thiamine) [mg]	1,8392	0,9035	0,0000	0,6192	0,0000	0,3165
Vitamin B2 (riboflavin) [mg]	1,9624	0,9484	0,0000	0,5425	0,0000	0,4715
Niacyna [mg]	21,1952	6,9769	0,0000	9,4664	0,0000	4,7519
Vitamin C [mg]	180,8365	43,0866	0,0000	110,1373	0,0000	27,6126
Vitamin A [micro g]	1718,2418	266,1492	0,0000	1206,5086	0,0000	245,5840
Vitamin E [mg]	21,5136	11,9359	0,0000	7,4198	0,0000	2,1579
Vitamin B6 [mg]	2,3429	0,7040	0,0000	1,2872	0,0000	0,3517
Vitamin B12 [µg]	1,8808	1,0120	0,0000	0,2388	0,0000	0,6300

vitamin D [micro g]	2,2160	0,0600	0,0000	1,3430	0,0000	0,8130
Glucose [g]	14,4766	5,6310	0,0000	7,6179	0,0000	1,2277
Saccharose [g]	17,4438	10,0537	0,0000	6,6600	0,0000	0,7301
Fructose [g]	15,3507	3,6181	0,0000	10,9477	0,0000	0,7849
Lactose [g]	9,3200	6,4000	0,0000	0,0000	0,0000	2,9200
Starch [g]	158,5747	73,8736	0,0000	43,4066	0,0000	41,2945
Dietary fibre [g]	45,9904	20,1346	0,0000	16,4855	0,0000	9,3703
Folin [g]	517,0704	207,5310	0,0000	193,9634	0,0000	115,5760
Cholesterol [mg]	95,7555	16,0000	0,0000	67,1555	0,0000	12,6000
Fatty acids: total saturated [g]	15,1844	6,8801	0,0000	3,5281	0,0000	4,7762
Fatty acids: total monounsaturated [g]	22,8710	8,4763	0,0000	10,4130	0,0000	3,9817
Fatty acids: 18:2 [g]	18,8371	11,8734	0,0000	4,2814	0,0000	2,6823
Fatty acids: 18:3 [g]	2,5283	0,8165	0,0000	1,2034	0,0000	0,5084
Fatty acids: total polyunsaturated [g]	21,3811	12,6899	0,0000	5,5005	0,0000	3,1907
Acids n-3 [g]	2,5283	0,8165	0,0000	1,2034	0,0000	0,5084
Acids n-6 [g]	18,8461	11,8734	0,0000	4,2904	0,0000	2,6823
Salt [g]	10,6083	2,3962	0,0000	6,8841	0,0000	1,3280
Water [ml]	2335,5624	759,0274	0,0000	1096,4252	0,0000	480,1098
% of energy from protein	20,35					
% of energy from fat	28,27					
% of energy from carbohydrates adsorbable	46,99					
% energy from fibre	4,39					
	51,38					

Menu for: METABOLIC MODEL DIET - 52,94 years per day 27.11.2022

Meal	Hour	Quantity	Product/dish
Breakfast	8:0	50,00	Boiled eggs [g]
Breakfast	8:0	10,00	Margarine "Rama" [g]
Breakfast	8:0	100,00	Tomato [g]
Breakfast	8:0	10,00	Chives [g]
Breakfast	8:0	250,00	Tea, infusion without sugar [ml, g]
Breakfast	8:0	50,00	Oatmeal [g]
Breakfast	8:0	10,00	Walnuts [g]
Breakfast	8:0	20,00	Sunflower, shelled seeds [g]
Breakfast	8:0	150,00	Apple [g]
Breakfast	8:0	100,00	Bread Graham [g]
Breakfast	8:0	100,00	Cottage cheese, grainy [g]
Breakfast	8:0	200,00	Yogurt natural 2% fat [g]
Dinner	12:0	100,00	Cooked lentils [g]
Dinner	12:0	50,00	Carrot [g]
Dinner	12:0	150,00	Pasta eggless cooked [g]
Dinner	12:0	10,00	Universal rapeseed oil [g]
Dinner	12:0	150,00	Cooked broccoli [g]
Dinner	12:0	250,00	Tea, infusion without sugar [ml, g]
Dinner	12:0	150,00	Tangerines [g]
Dinner	12:0	150,00	Tomato [g]
Dinner	12:0	330,00	Cauliflower soup with potatoes [g]
Supper	17:0	50,00	Pickled cucumber [g]
Supper	17:0	10,00	Margarine 'Rama" [g]
Supper	17:0	250,00	Tea, infusion without sugar [ml, g]
Supper	17:0	100,00	Bread Graham [g]
Supper	17:0	100,00	Turkey ham [g]
Supper	17:0	50,00	Red pepper [g]

Summary of the nutritional values of the menu:

Nutrient	of 24 hours	Breakfast	Lunch	Dinner	Tea	Supper
Energy [kcal]	1999,2273	985,4750	0,0000	620,3723	0,0000	393,3800
Energy % from a meal (without eating)		49,29	0,00	31,03	0,00	19,68
Total protein [g]	102,0094	48,1144	0,0000	27,8665	0,0000	26,0285
Fat [g]	60,9530	36,9742	0,0000	13,4803	0,0000	10,4985
Total carbohydrates [g]	280,7895	122,6403	0,0000	105,5582	0,0000	52,5910
Isoleucine [mg]	4742,4653	2376,7280	0,0000	1190,5423	0,0000	1175,1950
Leucine [mg]	7646,1815	3883,4170	0,0000	1918,2795	0,0000	1844,4850
Lysine [mg]	6276,2351	2626,0790	0,0000	1656,1811	0,0000	1993,9750
Methionine [mg]	2120,8491	1135,7720	0,0000	360,3271	0,0000	624,7500
Cystine [mg]	1589,3897	864,0620	0,0000	371,3477	0,0000	353,9800
Phenylalanine [mg]	4570,8845	2261,8320	0,0000	1293,0025	0,0000	1016,0500
Tryptophan [mg]	1209,7358	580,7570	0,0000	317,2338	0,0000	311,7450
Sodium [mg]	4591,5066	1054,3630	0,0000	2123,1486	0,0000	1413,9950
Potassium [mg]	4571,5957	1603,1630	0,0000	2183,0377	0,0000	785,3950
Calcium [mg]	806,6454	554,2130	0,0000	200,0274	0,0000	52,4050
Phosphorus [mg]	1889,3155	1080,4230	0,0000	399,6575	0,0000	409,2350
Magnesium [mg]	545,3290	268,2540	0,0000	153,1800	0,0000	123,8950
Iron [mg]	17,9062	7,5022	0,0000	6,9230	0,0000	3,4810
Zinc [mg]	14,7303	6,9412	0,0000	3,7152	0,0000	4,0739
Copper [mg]	2,1844	1,0853	0,0000	0,7675	0,0000	0,3316
Mangan [mg]	10,5705	5,2645	0,0000	2,9339	0,0000	2,3721
Retinol [µg]	344,8800	242,4000	0,0000	0,0000	0,0000	102,4800
Vitamin B1 (thiamine) [mg]	2,0643	0,9949	0,0000	0,7707	0,0000	0,2987
Vitamin B2 (riboflavin) [mg]	2,0810	1,1975	0,0000	0,5460	0,0000	0,3375
Niacin [mg]	23,8495	7,7113	0,0000	5,0884	0,0000	11,0498
Vitamin C [mg]	265,1602	38,0640	0,0000	163,8962	0,0000	63,2000
Vitamin A [micro g]	1985,9942	408,0963	0,0000	1242,5179	0,0000	335,3800
Vitamin E [mg]	20,7785	10,6215	0,0000	7,1253	0,0000	3,0317

Vitamin B6 [mg]	2,5543	0,7945	0,0000	1,0341	0,0000	0,7257
Vitamin B12 [µg]	3,1347	2,3187	0,0000	0,0000	0,0000	0,8160
vitamin D [micro g]	2,4663	1,6395	0,0000	0,0000	0,0000	0,8268
Glucose [g]	11,0640	3,4043	0,0000	6,7672	0,0000	0,8925
Saccharose [g]	20,3322	5,8213	0,0000	14,0724	0,0000	0,4385
Fructose [g]	14,9925	7,4242	0,0000	6,5483	0,0000	1,0200
Laktoza [g]	9,7800	9,7400	0,0000	0,0000	0,0000	0,0400
Starch [g]	175,2798	75,2065	0,0000	58,7808	0,0000	41,2925
Dietary fibre [g]	39,3922	14,9070	0,0000	16,9852	0,0000	7,5000
Folin [g]	559,6564	209,8843	0,0000	262,0921	0,0000	87,6800
Cholesterol [mg]	243,2800	187,6000	0,0000	0,0000	0,0000	55,6800
Fatty acids: total saturated [g]	15,2820	10,8448	0,0000	1,2257	0,0000	3,2115
Fatty acids: total monounsaturated [g]	20,6975	10,3889	0,0000	6,7483	0,0000	3,5603
Fatty acids: 18:2 [g]	17,9127	11,9225	0,0000	3,0523	0,0000	2,9379
Fatty acids: 18:3 [g]	2,4603	0,8348	0,0000	1,2146	0,0000	0,4109
Fatty acids: total polyunsaturated [g]	20,4519	12,8006	0,0000	4,2737	0,0000	3,3776
Acids n-3 [g]	2,4951	0,8696	0,0000	1,2146	0,0000	0,4109
Acids n-6 [g]	17,9502	11,9312	0,0000	3,0523	0,0000	2,9667
Salt [g]	11,4853	2,6377	0,0000	5,3109	0,0000	3,5367
Water [ml]	2375,7912	783,3920	0,0000	1138,5822	0,0000	453,8170
% of energy from protein	20,59					
% of energy from fat	27,03					
% of energy from carbohydrates adsorbable.	48,63					
% energy from fibre	3,75					
	52,38					

Menu for: METABOLIC MODEL DIET - 52.94 years per day 28.11.2022

Meal	Hour	Quantity	Product/dish
Breakfast	8:0	10,00	Margarine "Rama" [g]
Breakfast	8:0	250,00	Tea, infusion without sugar [ml, g]
Breakfast	8:0	150,00	Frozen strawberries [g]
Breakfast	8:0	20,00	Butter lettuce [g]
Breakfast	8:0	100,00	Tomato [g]
Breakfast	8:0	30,00	Sunflower, shelled seeds [g]
Breakfast	8:0	100,00	Bread Graham [g]
Breakfast	8:0	230,00	Milk, 2% fat [ml]
Breakfast	8:0	100,00	Cooked millet groats [g]
Breakfast	8:0	60,00	Cheese light [g]
Dinner	12:0	330,00	Cucumber soup [g]
Dinner	12:0	150,00	Soybean stew [g]
Dinner	12:0	5,00	Universal rapeseed oil [g]
Dinner	12:0	150,00	Green Bean [g]
Dinner	12:0	150,00	White cabbage and carrot salad [g]
Dinner	12:0	5,00	Universal rapeseed oil [g]
Dinner	12:0	250,00	Tea, infusion without sugar [ml, g]
Dinner	12:0	150,00	Apple [g]
Dinner	12:0	150,00	Barley groats, Cooked
Supper	17:0	10,00	Margarine "Rama" [g]
Supper	17:0	100,00	Cucumber [g]
Supper	17:0	250,00	Tea, infusion without sugar [ml, g]
Supper	17:0	100,00	Turkey ham [g]
Supper	17:0	100,00	Bread Graham [g]

Summary of the nutritional values of the menu:

Nutrient	of 24 hours	Breakfast	Lunch	Dinner	Tea	Supper
Energy [kcal]	2003,9393	928,9978	0,0000	691,0815	0,0000	383,8600
Energy % from a meal (without eating)		46,36	0,00	34,49	0,00	19,16
Total protein [g]	99,6564	46,1784	0,0000	27,9980	0,0000	25,4800
Fat [g]	69,4843	38,4402	0,0000	20,7361		0,0000
10,3080						
Total carbohydrates [g]	266,2550	106,6525	0,0000	108,6785	0,0000	50,9240
Isoleucine [mg]	4540,2269	2121,5020	0,0000	1265,2649	0,0000	1153,4600
Leucine [mg]	7389,7846	3485,3201	0,0000	2096,0245	0,0000	1808,4400
Lysine [mg]	6195,5580	2615,2570	0,0000	1616,3010	0,0000	1964,0000
Methionine [mg]	1970,2306	961,6910	0,0000	391,1796	0,0000	617,3600
Cystine [mg]	1397,1495	583,6940	0,0000	473,0955	0,0000	340,3600
Phenylalanine [mg]	4393,1976	2072,6700	0,0000	1325,6676	0,0000	994,8600
Tryptophan [mg]	1305,9708	551,7410	0,0000	451,5898	0,0000	302,6400
Sodium [mg]	4218,5109	1161,3350	0,0000	1988,0359	0,0000	1069,1400
Potassium [mg]	4454,6247	1464,0530	0,0000	2273,0517	0,0000	717,5200
Calcium [mg]	1195,2093	836,5966	0,0000	309,9327	0,0000	48,6800
Phosphorus [mg]	2045,9966	1055,2012	0,0000	591,6754	0,0000	399,1200
Magnesium [mg]	601,5178	280,4180	0,0000	217,6198	0,0000	103,4800
Iron [mg]	18,8670	7,4879	0,0000	8,2091	0,0000	3,1700
Zinc [mg]	15,0254	7,0223	0,0000	4,2769	0,0000	3,7262
Copper [mg]	2,7839	1,3156	0,0000	1,1215	0,0000	0,3468
Mangan [mg]	9,6463	4,0439	0,0000	3,2652	0,0000	2,3372
Retinol [µg]	348,7270	246,2470	0,0000	0,0000	0,0000	102,4800
Vitamin B1 (thiamine) [mg]	1,8537	1,0562	0,0000	0,4999	0,0000	0,2976
Vitamin B2 (riboflavin) [mg]	1,7571	1,0697	0,0000	0,3835	0,0000	0,3039
Niacin [mg]	23,2738	8,7589	0,0000	4,1083	0,0000	10,4066
Vitamin C [mg]	189,8443	114,0966	0,0000	69,9877	0,0000	5,7600
Vitamin A [micro g]	1454,2062	394,2930	0,0000	937,2732	0,0000	122,6400
Vitamin E [mg]	19,3605	11,8370	0,0000	5,6091	0,0000	1,9144
Vitamin B6 [mg]	2,3805	0,9325	0,0000	0,8848	0,0000	0,5632
Vitamin B12 [µg]	2,2040	1,3880	0,0000	0,0000	0,0000	0,8160

vitamin D [micro g]	1,7226	0,8958	0,0000	0,0000	0,0000	0,8268
Glucose [g]	11,5246	4,8156	0,0000	6,2050	0,0000	0,5040
Saccharose [g]	13,1461	2,9122	0,0000	9,7659	0,0000	0,4680
Fructose [g]	15,5463	5,1155	0,0000	9,9268	0,0000	0,5040
Lactose [g]	11,2743	11,2343	0,0000	0,0000	0,0000	0,0400
Starch [g]	161,7382	64,0060	0,0000	56,4602	0,0000	41,2720
Dietary fibre [g]	41,4734	13,2516	0,0000	21,4618	0,0000	6,7600
Folin [g]	434,9688	165,7690	0,0000	196,8198	0,0000	72,3800
Cholesterol [mg]	103,7920	48,1120	0,0000	0,0000	0,0000	55,6800
Fatty acids: total saturated [g]	18,6474	13,1973	0,0000	2,2745	0,0000	3,1756
Fatty acids: total monounsaturated [g]	22,0640	10,3370	0,0000	8,1752	0,0000	3,5518
Fatty acids: 18:2 [g]	21,7458	12,1413	0,0000	6,7387	0,0000	2,8658
Fatty acids: 18:3 [g]	2,7752	0,7363	0,0000	1,6661	0,0000	0,3728
Fatty acidsEC: total polyunsaturated [g]	24,5558	12,8770	0,0000	8,4114	0,0000	3,2674
Acids n-3 [g]	2,7752	0,7363	0,0000	1,6661	0,0000	0,3728
Acids n-6 [g]	21,7746	12,1413	0,0000	6,7387	0,0000	2,8946
Salt [g]	10,5540	2,9069	0,0000	4,9726	0,0000	2,6745
Water [ml]	2414,8579	846,9665	0,0000	1131,2734	0,0000	436,6180
% of energy from protein	20,03					
% of energy from fat	30,82					
% of energy from carbohydrates is consumed	45,21					
% energy from fibre	3,94					
	49,15					

Average nutritional values from 5 menus for: METABOLIC MODEL DIET from 24.11.2022 to 28.11.2022

Nutrient	implementation of standards	% EAR	% RDA	% AI
Energy [kcal]	2005,3610	91,15		
Total protein [g]	104,6252	260,59	211,36	
Fat [g]	60,9040	83,43	83,43	
Total carbohydrates [g]	281,0946			
Isoleucine [mg]	4895,3143			
Leucine [mg]	7799,8541			
Lysin [mg]	6762,5348			
Methionine [mg]	2219,3537			
Cystine [mg]	1565,4112			
Phenylalanine [mg]	4568,7365			
Tryptophan [mg]	1348,5633			
Sodium [mg]	4367,6367			291,18
Potassium [mg]	4765,3688			136,15
Calcium [mg]	899,4576	112,43	89,95	
Phosphorus [mg]	2067,6234	356,49	295,37	
Magnesium [mg]	626,8001	179,09	149,24	
Iron [mg]	19,4926	324,88	194,93	
Zinc [mg]	15,0159	159,74	136,51	
Copper [mg]	2,4525	350,35	272,50	
Mangan [mg]	9,7614			424,41
Retinol [µg]	255,0142			
Vitamin B1 (thiamine) [mg]	1,8994	172,67	146,11	
Vitamin B2 (riboflavin) [mg]	1,9094	173,58	146,88	
Niacin [mg]	25,8110	215,09	161,32	
Vitamin C [mg]	226,0619	301,42	251,18	
Vitamin A [micro g]	1694,4458	268,96	188,27	
Vitamin E [mg]	20,5125			205,12
Vitamin B6 [mg]	2,7654	197,53	162,67	
Vitamin B12 [µg]	2,7001	135,01	112,51	
vitamin D [micro g]	2,1766			14,51
Glucose [g]	12,0433			
Saccharose [g]	17,2519			
Fructose [g]	15,5311			
Lactose [g]	10,1457			
Starch [g]	171,3747			
Dietary fibre [g]	42,6797			170,72
Folin [g]	504,5015	157,66	126,13	
Cholesterol [mg]	178,6071			
Fatty acids: total saturated [g]	14,5558			108,63
Fatty acids: total monounsaturated [g]	21,2539			
Fatty acids: 18:2 [g]	18,2510			186,66
Fatty acids: 18:3 [g]	2,5607			213,39
Fatty acids: total polyunsaturated [g]	20,9231			
Acids n-3 [g]	2,6319			
Acids n-6 [g]	18,2831			
Salt [g]	10,9260			
Water [ml]	2274,2397			90,97
% of energy from protein	21,07			
% of energy from fat	26,96			
% of energy from carbohydrates adsorbable.	47,92			
% energy from fibre	4,05			
% of energy from total carbohydrates	51,97			

Reference % of energy from total carbohydrates (digestible + fibre): 45%-65%
